# Automated high throughput IgG N-glycosylation sample preparation method development on the Tecan Freedom EVO platform

**DOI:** 10.11613/BM.2024.020708

**Published:** 2024-06-15

**Authors:** Borna Rapčan, Maja Hanić, Branimir Plavša, Jelena Šimunović, Jerko Štambuk, Frano Vučković, Irena Trbojević-Akmačić, Mislav Novokmet, Gordan Lauc, Genadij Razdorov

**Affiliations:** 1 Faculty of Pharmacy and Biochemistry, University of Zagreb, Zagreb, Croatia; 2 Genos Glycoscience Research Laboratory, Zagreb, Croatia

**Keywords:** laboratory automation, immunoglobulin G, glycomics, chromatography, validation/evaluation

## Abstract

**Introduction:**

Glycomics, focusing on the role of glycans in biological processes, particularly their influence on the folding, stability and receptor interactions of glycoconjugates like antibodies, is vital for our understanding of biology. Changes in immunoglobulin G (IgG) N-glycosylation have been associated with various physiological and pathophysiological conditions. Nevertheless, time-consuming manual sample preparation is one of the limitations in the glycomics diagnostic implementation. The study aimed to develop an automated method for sample preparation on the Tecan Freedom Evo 200 platform and compare its efficiency and precision with the manual counterpart.

**Materials and methods:**

The initial method development included 32 pooled blood plasma technical replicates. An additional 24 pooled samples were used in the method comparison along with 78 random duplicates of plasma samples collected from 10,001 Dalmatians biobank to compare the manual and automated methods.

**Results:**

The development resulted in a new automated method. For the automated method, glycan peaks comprising 91% of the total sample glycan showed a variation of less than 5% while 92% of the total sample showed a variation of less than 5% for the manual method. The results of the Passing-Bablok regression indicated no differences between the automated and manual methods for 12 glycan peaks (GPs). However, for 8 GPs systematic difference was present, while both systematic and proportional differences were present for four GPs.

**Conclusions:**

The developed automated sample preparation method for IgG glycan analysis reduced exposure to hazardous chemicals and offered a simplified workflow. Despite slight differences between the methods, the new automated method showed high precision and proved to be highly comparable to its manual counterpart.

## Graphical abstract

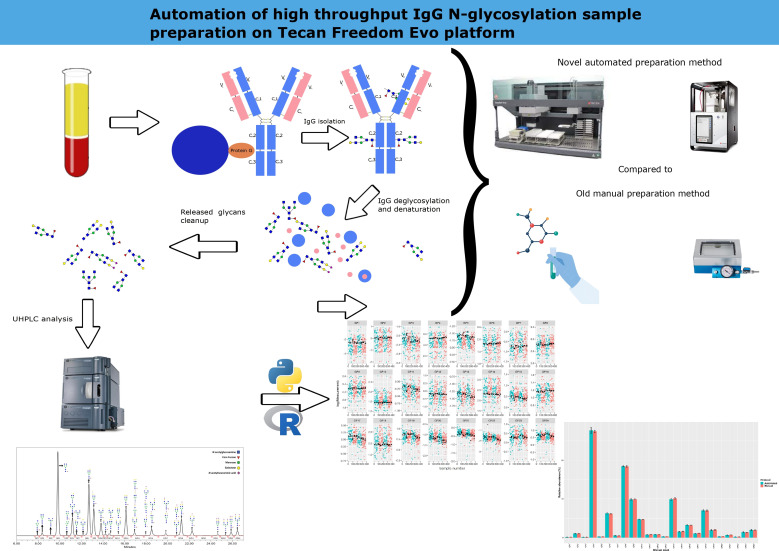

## Highlights

• An automated immunoglobulin G (IgG) N-glycan method was developed leveraging positive pressure technology

• In general, automated method showed satisfactory precision and agreement with manual method that is in current use

• The automated method holds the possibility to significantly streamline sample preparation, enhancing throughput and improving laboratory safety

## Introduction

The term “glycomics” refers to a diverse range of scientific methods and techniques that are utilized to identify and measure all the sugar molecules (known as glycans) that are attached to other complex biological molecules, produced by cells, tissues or organisms under specific conditions of time, location and environment. Glycosylation is the most common protein posttranslational modification of proteins (*1*). Therefore, it is no surprise that its research has quickly grown in importance in the last decade, with myriads of research efforts focused on discovering the importance of these complex molecular structures. The research effort resulted in discoveries that glycans influence glycoconjugate folding, stability and receptor interaction (*2*-*4*). Posttranslational modifications of immunoglobulin G (IgG), such as N-glycosylation, play a significant role in antibody activity and functions (*5*). Immunoglobulin G N-glycosylation was implicated in numerous physiological processes such as ageing, weight variation, lifestyle interventions and hormonal oscillation (*6*, *7*). Changes were also observed in pathological conditions, including different types of cancer, diabetes, neurological disorders and infectious diseases among other examples (*8*-*11*). Glycosylation is critical for the biopharmaceutical efficacy and safety profile of therapeutic antibodies (*12*). Due to growing knowledge of protein glycosylation, the need for novel sample preparation and analytical tools has been rapidly emerging (*13*).

Methods for IgG glycan analysis typically adhere to a standardized sequence. This sequence starts with the purification of IgG samples *via* affinity purification utilizing solid-supported Protein A or Protein G (*13*). Following this, the concentrated protein undergoes denaturation, and the glycans are released with the assistance of PNGase F. Subsequently, glycan labelling ensues. The glycans are labelled, separated from proteins and excess reagents, and analysed *via* capillary gel electrophoresis (CGE) or high- or ultra-high-performance liquid chromatography (HPLC or UHPLC) (*14*).

Broad clinical trials and genome-wide association studies can give a much more in-depth insight into the functional relevance of glycosylation and its regulation and role in diverse conditions. Studies of this type require a large number of samples to be analysed in a short time, driving the need for the development of high-throughput methodologies for glycosylation analysis (*14*). Adopting automated methods would greatly streamline glycome in epidemiology and clinical diagnostics, enhancing the rapid discovery of glycan biomarkers (*15*). A study by Groth and Cox found that in 2017, 89% of published life science articles featured a manual protocol with an existing automated alternative (*16*). The second advantage is that laboratory automation helps eliminate most human errors, yielding more reliable results. According to a study conducted by Szecsi *et al.* in Denmark, approximately 80% of the errors that occur in a clinical biochemistry laboratory can be attributed to human error (*17*). Automated systems allow for more sophisticated pipetting techniques. The digital nature of protocols means effortless transfer between laboratories, making the knowledge more accessible. Lastly, automation eliminates complicated and tedious analysts’ tasks, reduces the risk of exposure to hazardous materials, frees up human resources and lowers costs (*15*).

These benefits come with particular challenges. The biggest challenge is the complexity of methods used in glycomics (*15*). Pipetting systems, while very precise, come with a set of limitations of their own. Issues with “dead” volumes often appear, increasing the cost of consumables needed for the sample preparation. Another problem can be connected to sample stability and evaporation. Often, samples must be covered and kept at the right temperature, which is challenging to automate. Novel instruments allow manoeuvring of lids and temperature control. However, there are still steps in the protocol when this takes longer to achieve compared to manual methods. Robots are also challenging to control as they require additional knowledge of instrument software. The last challenge with introducing such systems in laboratories is the exorbitant prices, that make it hard for many research teams to acquire such a device (*15*).

Multipurpose liquid-handling robotic workstations have been designed to automate much of the sampling, mixing and manipulating of liquid samples. The two main types of automated liquid-handling workstations used in glycomics laboratories fit into one of the two robotic configurations: Cartesian configuration (moves in three perpendicular axes (X, Y and Z) that allow precise movements and positioning of the robotic arm) and the anthropomorphic or articulated configuration (human-like or arm-like configuration with multiple joints for movement) (*15*). There were attempts to develop methods for antibody glycan analysis using Biomek FX manufactured by Beckman Coulter, using MICROLAB STARlet developed by Hamilton, Andrew Alliance semi-automation platform and SweetBlot 7 automated system from System Instruments Co. (*18*-*23*).

A hypothesis occurred that automatization would minimize dependence on analyst interventions and mitigate the current drawbacks of the manual method. Therefore, this study aimed to develop an automated method for sample preparation on the Tecan Freedom Evo 200 platform and compare its efficiency and repeatability with the manual counterpart (*14*).

## Materials and methods

### Subjects

For this study, 32 pooled samples of healthy donors along with 8 blanks (void of sample, containing only ultrapure water) recruited at the Croatian Institute of Transfusion Medicine (CITM) were used for initial method evaluation as technical replicates. The use of these samples for research purposes was allowed by the Ethics Committee of the CITM. For the comparison between the manual and automated methods plasma samples were collected from 10,001 Dalmatians biobank (*24*, *25*). The sample set for the comparison included 78 duplicate plasma samples, along with additional 24 samples of pooled donors from the CITM and 12 blanks. No demographic, anamnestic or clinical data, collected for the establishment of the 10,001 Dalmatian Biobank, were used in this study. Blood samples were collected by venipuncture into the vacuum tubes with dipotassium salt of ethylenediaminetetraacetic acid (Beckton Dickinson, Franklin Lakes, USA).

## Methods

### Sample randomisation

Sample randomisation was performed using a simple Python script (*26*). The script used the pre-made list of 78 duplicate samples from 10,001 Dalmatian Biobank, shuffled them using the Python random function (with no predetermined rules) and distributed them to two 96-well plates. The program then added an equal number of positions for blanks and plasma pool to each plate. Six blanks (12 in total) and 12 plasma pools (24 in total) positions were added to each plate. They were shuffled again to randomise all positions. The same plate layout was used for both the automatic and manual approaches. Plasma samples separated by centrifugation at room temperature for 10 minutes at 1500xg and stored at - 20 °C for up to 15 years were unfrozen and randomised per generated plate layouts. These plate layouts were pipetted on the same day and subsequently frozen at - 20 °C. Before executing each IgG isolation, the frozen plates used for manual and automated analysis were thawed and processed in parallel.

### Tecan EVO 200 composition

For this study, we used the Tecan Freedom Evo 200 platform in combination with the Resolvex A200 positive pressure unit. The robot worktable was equipped with various components, including the Te-Shake Option 2 for shaking with heating, two CPAC Tec-Control devices (modified to hold plates and tubes, respectively by the manufacturer) and an incubator 4-slot MIO2 60 °C module. Additionally, the worktable had five plate carriers (two of which were altered by the manufacturer), a hanging rack for disposable tips and a shelf for labware. Two racks for throughs to hold liquids and two Tecan Hotel Decks were also present. Tecan had two arms, one for pipetting liquids (the Liquid Handling Arm or LiHa) and the other for labware manipulation (the Robot Manipulator Arm or RoMa). The A200 was fitted with a tall plate stand, a positive pressure manifold, and a 8-channel pump for liquid dispensing.

The automated platform was controlled using Tecan Freedom EVOware software and A200 control software. These softwares were used to write custom methods to be executed by the platform. Freedom EVOware software manipulated both devices and started methods written using A200 software while performing its scripts, enabling parallel operations on both machines. The automated method was designed to imitate the existing manual UHPLC sample preparation method (*15*, *27*).

### Manual IgG isolation

**Figure 1:**
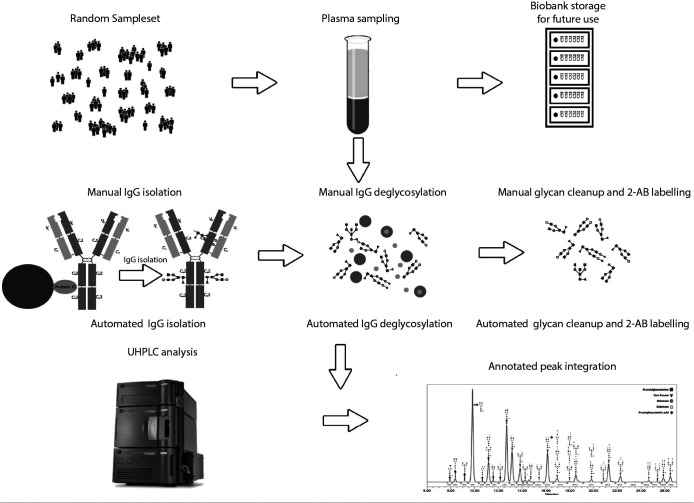
The flowchart illustrates the sequential processes involved in the analysis. The first row outlines the subject recruitment and sampling protocol, followed by the analysis preparation protocol in the second row. The third row represents the steps of ultra-high-performance liquid chromatography (UHPLC) analysis and peak integration.

For IgG isolation, we followed previously reported protocols (*14*, *27*, *28*). A plasma sample of 100 μL or blank was pipetted into each well of the plate and diluted in a ratio of 1:7 with 1x phosphate-buffered saline (1xPBS, 0.137M NaCl, 0.0097M Na_2_HPO_4_, 0.0022M KH_2_PO_4_, 0.0027M Na_2_HPO_4_, pH = 7.4). Following filtration through a 0.45 μm wwPTFE 96-well plate (Pall Corporation, New York, USA) on a vacuum manifold under the pressure of 58.6 kPa, filtrates were transferred onto CIM r-Protein G LLD 0.2 mL Monolithic 96-well Plate (2 µm channels) (Sartorius, Göttingen, Germany). An additional filtration step was employed on the vacuum manifold and combined with washing three times with 2 mL 1xPBS. Next, the bound IgG was released with 1 mL of 0.1 M formic acid under the pressure of 34.5 kPa and neutralized with 0.17 mL of 1 M ammonium bicarbonate. Finally, 300 µL of the eluted sample was pipetted onto a 1 mL plate and set aside for deglycosylation, while the remaining volume was manually removed and frozen at - 20 °C (Figure 1).

The major difference from the manual method was that a vacuum manifold (Pall Corporation, New York, USA) was used to perform actions on protein G and wwPTFE filter plates.

### Automated IgG isolation

The automated protocol was designed to mimic the manual protocol closely. It starts in the same way diluting the plasma with 1xPBS. Following the dilution first major difference occurs. Filtration of diluted plasma, including all subsequent filtration steps, was performed using the A200 (Tecan, Männedorf, Switzerland) positive pressure unit using an automated profile. The automated protocol employs a new 10-minute filtration profile that was programmed where the pressure increased from 55 kPa to 165 kPa, culminating in a short burst of high pressure 220 kPa during the final five seconds. After plasma filtration, the automated protocol follows the same pattern as its manual counterpart.

### Manual deglycosylation

Previously isolated 300 μL IgG samples were dried in a Savant SpeedVac (Thermo Fisher, Waltham, USA) (*14*). The dried IgG samples were resuspended manually with 30 µL of 1.33% sodium dodecyl sulphate (SDS, Sigma-Aldrich, St. Louis, USA) and denatured at 65 °C for 10 minutes. After resuspension, 10 µL of 4% Igepal-CA630 (Sigma-Aldrich, St. Louis, USA) was added, resuspended and incubated at room temperature for 15 min. N-glycans were released with 1.2 units of recombinant N-glycosidase F (Promega, Madison, USA) during an 18 h incubation at 37 °C (Figure 1).

### Automated deglycosylation

Following IgG isolation drying and SDS and resuspension steps are performed identically to the manual method. The first major difference occurs during the denaturation step where due to equipment limitations incubation is performed at 60 °C for 10 minutes. The rest of the automated method follows the manual method with the resuspension steps of the manual method being replaced with vigorous shaking using the Te-Shake module at a speed of 1000 rpm.

### Manual glycan labelling and cleanup

Previously released N-glycans were labelled with 25 μL of fluorescent dye, 2-aminobenzamide (2-AB) (Sigma-Aldrich, St. Louis, USA), in a reductive amination reaction with 2-picoline borane (2-PB) (Sigma-Aldrich, St. Louis, USA) and incubated for 2 hours at 65 °C (*14*). Glycan cleanup was performed by hydrophilic interaction liquid chromatography solid phase extraction using a 0.2 μm wwPTFE 96-well filter plate (Pall Corporation, New York, USA) using the vacuum manifold. The cleanup part of the protocol starts with filter plate preconditioning washing the plate with 200 μL of 70% EtOH, followed by 200 μL of ultrapure water and 200 μL of 96% ACN. This is then followed by transferring the 2-AB labelled glycans onto the preconditioned wwPTFE filter plate along with 700 μL 100% cold ACN. This mixture is resuspended and incubated for 2 minutes. After this, it is washed 3 times with 200 μL of 96% ACN. Finally, the glycans are eluted using 180 μL of ultrapure water and collected into a clean 0.8ml round well plate (Waters, Milford, USA) (Figure 1).

### Automated glycan labelling and cleanup

Much like the previous steps the automated protocol follows its manual counterpart. The main difference was that the cleaning steps were performed using A200 using a specially programmed pressure profile. The incubation temperature was reduced to 60 °C just like in the deglycosylation protocol. A specially designed, in-house 3D-printed collar was also introduced for the filter plate to overcome height constraints and enable its use on A200, to maintain filter integrity and to prevent cross contamination. The collection plates were replaced with square well 1 mL short plates (Thermo Fisher, Waltham, USA) to allow automated plate sandwiching.

### Ultra-high-performance liquid chromatography analysis

**Figure 2:**
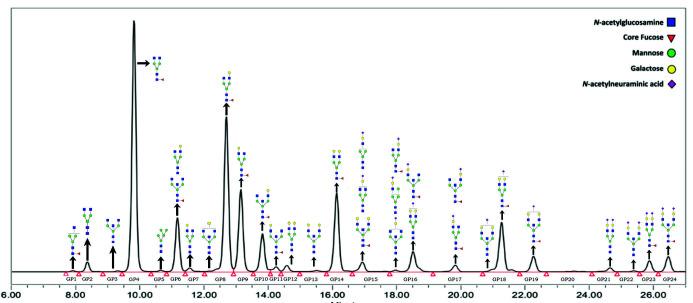
Annotated and integrated chromatogram of glycans separated by hydrophilic interaction liquid chromatography - ultra-high-performance liquid chromatography (HILIC-UHPLC) analysis of the IgG glycome. Raw glycan intensities are computed as areas under the curve of the corresponding chromatographic peak.

The prepared samples were stored for up to 2 weeks at - 20 °C until UHPLC analysis on Waters Acquity H class UPLC instrument (Waters, Milford, USA). 100 mM ammonium formate in water (pH = 4.4) was used as solvent A, and acetonitrile was used as solvent B. A volume of 40 μL of labelled IgG N-glycans was separated on a 100 mm Glycan BEH Amide column (Waters, Milford, USA) at 10 °C with a linear gradient of 75-62% solvent B. Flow rate was kept at 0.4 mL/min during the 29 min gradient. Samples were detected with ACQUITY Premier FLR Detectors at 360 nm excitation wavelength, and 425 nm emission wavelength. Peak data were integrated manually using Empower 3 software (Waters, Milford, USA) dividing each chromatogram into 24 separate IgG glycan peaks (labelled as GPs followed by the number in the order in which they appear in the chromatogram) with known structures (Figure 2). The amount of glycan contained in each peak was expressed as RA (relative abundance, a percentage of the total integrated area).

### Statistical analysis

The Python programming language, with the help of the Matplotlib (v.3.7.1), NumPy (v.1.24.2), Seaborn (v.0.12.2) and Pandas (1.5.3) libraries, was used to create figures and for the statistical analysis (*26*). Following the previously reported data for the precision of IgG N-glycan analysis methods we defined the following coefficient of variation (CV) acceptability criteria: 5% for peaks with RA exceeding 3%, 10% for peaks with RA 0.5-3%, and 15% for peaks with RA less than 0.5% (*20*, *29*). Testing of the agreement between automated and manual sample preparation protocols using the Passing-Bablok regression was performed with MedCalc software (v. 22.019, MedCalc, Ostend, Belgium) was used.

## Results

### Precision of the developed automated method

The automated method demonstrated satisfactory precision when analyzing the CITM plasma pool samples.

Table 1 presents the precision data obtained in the developmental phase. Analyzing 32 plasma pool technical replicates, CVs for multiple glycan peaks (GP4, GP6, GP8, GP9, GP10, GP14, GP16 and GP18, together comprising 85% of measured IgG glycome) consistently remained at 2% or lower. Only peaks with minor RA, such as GP5, GP13, and GP20 through GP22 (together comprising 1.75% of measured IgG glycome), exhibited CVs above 10%. No observable signals were detected in blanks, indicating a lack of cross-contamination. When comparing these results to previously described acceptance criteria, 21 were acceptable. Three peaks GP21, GP23 and GP24 showed higher CV values (Table 1).

**Table 1:** The precision of the automated method assessed in the developmental phase by analyzing 32 plasma pool technical replicates

**GP number**	**Average RA (%)**	**SD (%)**	**CV (%)**
1	0.07	0.00	5.88
2	0.34	0.03	7.68
3	0.19	0.02	7.84
4	21.20	0.41	1.95
5	0.15	0.02	12.69
6	4.57	0.08	1.81
7	0.18	0.01	7.60
8	17.50	0.32	1.83
9	10.83	0.17	1.57
10	3.99	0.04	0.91
11	0.67	0.02	3.22
12	0.45	0.04	7.84
13	0.11	0.01	11.49
14	13.07	0.24	1.82
15	1.49	0.04	2.47
16	3.85	0.07	1.87
17	0.92	0.06	6.93
18	10.22	0.20	2.00
19	2.61	0.19	7.32
20	0.25	0.03	13.29
21	1.10	0.16	14.59
22	0.14	0.02	13.79
23	3.03	0.25	8.19
24	3.08	0.27	8.74
GP- glycan peak. RA - relative abundance. SD - standard deviation. CV - coefficient of variation.			

**Figure 3:**
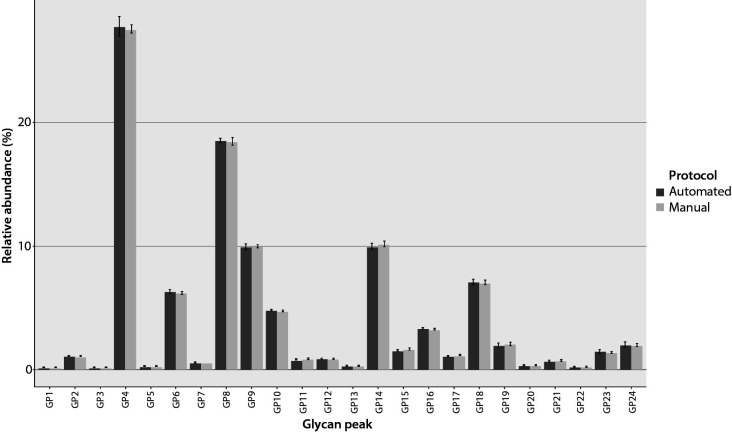
Average relative abundances (RAs) for each of the 24 glycan peaks (GP) of pooled plasma samples between the automated and manual protocols. The dark gray bars represent the average GP RA obtained using the Tecan workstation, while the light gray bars represent the average GP RA obtained using the manual protocol. Error bars illustrate the ± standard deviations (SDs), for each peak when the same plasma pool samples were analyzed using manual and automated protocols.

Table 2 shows the precision results obtained by analysing 24 plasma pool technical replicates in parallel with the method comparison sample set. As detailed in Table 2, CVs for GP3, GP8, GP11, GP12, GP15 and GP16 were lower in the automated than in the manual method. Specifically, when measured using the automated method, 11 GPs, or 91% of total measured glycome had CVs below 5%, whereas the manual method yielded 13 GPs, or 92% of total glycome, with CVs below 5%. In cases where CVs ranged from 5% to 10%, the automated method had eight such peaks amounting to 6.48% of total glycome, while the manual method had nine, totalling 8.16% of total glycome. Additionally, the automated method had four glycan peaks with CVs between 10% and 15%, together totalling 2.85% of total glycome, the majority of which was contributed by GP24 (1.96%), whereas the manual method had two, total glycome contributions equalling 0.3%. Notably, GP22 was the only glycan peak with a CV greater than 15% when prepared using the automated method. When comparing CVs with the corresponding acceptance criteria, results for 22 glycan peaks were acceptable. Two peaks, GP22 and GP24, had CVs for the automated method greater than the declared criteria. The bars in Figure 3 illustrate the RA average along with ± standard deviations (SDs) as error bars, for each peak when the same plasma pool samples were analysed using manual and automated protocols.

**Table 2:** The precision of manual and automated methods assessed by analyzing 24 plasma pool technical replicates

	**Average RA (%)**		**SD (%)**		**CV (%)**	
**GP number**	**Method**		**Method**		**Method**	
	**Automated**	**Manual**	**Automated**	**Manual**	**Automated**	**Manual**
1	0.11	0.11	0.01	0.01	8.28	8.09
2	1.04	1.02	0.06	0.05	5.39	4.91
3	0.11	0.12	0.01	0.01	7.58	11.38
4	27.72	27.49	0.76	0.33	2.73	1.21
5	0.19	0.21	0.02	0.01	10.08	6.58
6	6.27	6.16	0.15	0.11	2.34	1.85
7	0.50	0.47	0.05	0.03	9.31	5.69
8	18.47	18.39	0.18	0.30	0.95	1.65
9	9.88	9.92	0.21	0.11	2.17	1.15
10	4.75	4.68	0.07	0.06	1.42	1.21
11	0.75	0.82	0.04	0.06	5.45	7.37
12	0.81	0.82	0.02	0.03	3.04	4.20
13	0.25	0.26	0.03	0.02	11.75	8.36
14	9.91	10.1	0.25	0.22	2.54	2.18
15	1.51	1.61	0.05	0.08	3.41	4.88
16	3.27	3.2	0.06	0.07	1.78	2.34
17	1.04	1.12	0.05	0.05	4.60	4.51
18	7.04	7.00	0.22	0.18	3.12	2.50
19	1.91	2.02	0.17	0.13	8.86	6.47
20	0.28	0.31	0.03	0.02	10.34	6.22
21	0.65	0.69	0.06	0.06	9.05	8.35
22	0.17	0.18	0.03	0.02	16.50	12.09
23	1.41	1.33	0.13	0.05	9.14	3.49
24	1.96	1.94	0.21	0.11	10.70	5.77
GP - glycan peak. RA - relative abundance. SD - standard deviation. CV - coefficient of variation.						

### Comparison of manual and automated methods

The results of the Passing-Bablok regression (Table 3) indicated no differences between the automated and manual methods for 12 GPs. However, for 8 GPs systematic difference was present, while both systematic and proportional differences were present for four GPs. Comparison between the two methods indicated systemic error less or equal to 5%.

**Table 3:** Passing-Bablok regression data for 24 glycan peaks

**GP number**	**Regression equation**	**Cusum test for linearity**
1	y = 0.00 (0.00 to 0.00) + 1.00 (1.00 to 1.00) x	0.57
2	y = -0.00 (0.00 to 0.02) + 1.01 (0.97 to 1.00) x	0.85
3	y = 0.01 (- 0.02 to 0.01) + 1.00 (1.00 to 1.25) x	0.20
4	y = -0.49 (- 0.88 to - 0.17) + 1.01 (0.99 to 1.02) x	0.74
5	y = 0.01 (0.01 to 0.04) + 1.00 (0.80 to 1.00) x	0.61
6	y = - 0.12 (- 0.20 to - 0.03) + 1.01 (0.99 to 1.02) x	0.74
7	y = - 0.01 (- 0.01 to 0.01) + 0.99 (0.95 to 1.00) x	0.15
8	y = 0.36 (- 0.19 to 0.86) + 0.97 (0.94 to 1.00) x	0.74
9	y = - 0.41 (- 0.64 to - 0.20) + 1.04 (1.02 to 1.07) x	0.61
10	y = - 0.11 (- 0.04 to 0.04) + 1.01 (1.00 to 1.03) x	0.49
11	y = - 0.00 (- 0.04 to 0.04) + 1.05 (1.00 to 1.10) x	0.21
12	y = - 0.01 (0.01 to 0.05) + 1.05 (1.04 to 1.07) x	0.44
13	y = 0.01 (-0.21 to 0.07) + 1.00 (0.85 to 1.00) x	0.02
14	y = - 0.05 (0.04 to 0.15) + 1.03 (1.02 to 1.04) x	0.37
15	y = 0.10 (- 0.13 to 0.02) + 1.00 (0.97 to 1.04) x	0.37
16	y = - 0.06 (0.01 to 0.14) + 0.99 (0.97 to 1.02) x	0.49
17	y = 0.10 (- 0.07 to 0.18)+ 1.00 (0.96 to 1.09) x	0.37
18	y = 0.07 (0.05 to 0.33) + 0.99 (0.97 to 1.01) x	1.00
19	y = 0.18 (0.03 to 0.10) + 0.96 (0.88 to 1.04) x	0.05
20	y = 0.05 (0.00 to 0.19) + 0.93 (0.80 to 1.00) x	0.21
21	y = 0.10 (0.02 to 0.05) + 0.926 (0.80 to 1.06) x	0.49
22	y = 0.02 (- 0.07 to 0.07) + 1.00 (0.83 to 1.00) x	0.41
23	y = - 0.01 (- 0.06 to 0.06) + 0.96 (0.91 to 1.01) x	0.74
24	y = 0.31 (- 0.63 to - 0.17)+ 0.84 (1.09 to 1.33) x	0.48
GP - glycan peak.		

## Discussion

The automated IgG isolation method demonstrated high precision, although slightly lower than the manual method. Glycan peaks 19 through 24 experienced higher variations than earlier peaks with similar RAs (Table 2). However, this remains a known issue due to their structures containing N-Acetylneuraminic acid (Neu5Ac), which was previously reported to be less stable and thus more challenging to quantify (*27*).

For half of the IgG N-glycans, there was no difference between the results obtained with the automated and manual methods. The systematic difference was noted for one-third of them, while the combination of the systemic and proportional differences appeared for one-sixth of the IgG N-glycans. In the studies by Pučić *et al.* and later by Hanić *et al.*, the glycan peak values are initially normalized to represent 100% of the glycome for the entire chromatogram (*27*, *28*). Subsequently, the abundance of each peak is determined by calculating its ratio relative to the total contents of the chromatogram. As outlined in Skhunnikova *et al.*’s review, the majority of glycomic studies prioritize the analysis of glycan trait ratios over absolute quantities to differentiate between two or more cohorts (*30*). The presence of a systematic error indicates that all peaks would have a consistently higher or lower abundance when the manual method is compared with the automated, but their ratios will be unlikely altered. This justifies the standpoint that finding systematic error for 12 peaks, during the comparison between automatic and manual methods does not mitigate the reliability of the new method. This approach ensures that any systematic error that uniformly unlikely to alter the ratios. The notable exception was GP24, however, this finding may not be surprising due to the high content of Neu5Ac known for its instability during the analytical process (*27*). Our evaluation uncovered a persistent systemic error, while the absence of significant proportional differences in the majority of individual samples suggests a consistent but small systematic difference between methods. As a result, we confidently inferred that the methods exhibit high similarity, affirming their suitability and comparability for this specific purpose.

In the introduction, we presented several studies focusing on different approaches to automation in the field of glycomics. However, drawing direct comparisons between these methods is challenging due to the use of varied analytical techniques and analytes. Our approach introduces a high-throughput analysis of plasma IgG N-glycans utilizing UHPLC. While previous efforts have been made to develop automated methods for studying the human glycome, most reported methodologies did not prioritize the development of a high-throughput approach specifically for analyzing IgG glycome using UHPLC. Stöckmann *et al.* conducted a study with a similar overarching objective, which served as a valuable reference for establishing precision criteria for high-throughput IgG N-glycosylation analysis (*20*). It is noteworthy that the majority of glycan peaks in our method demonstrate lower CVs compared to those reported in Stöckmann *et al.*’s study (*20*). This underscores the enhanced precision of our new approach and represents a notable advancement over previous methodologies. Furthermore, our approach benefits from a significantly larger sample size used to assess method precision. Additionally, we conducted a comprehensive comparison with the manual method from which our automated approach was derived which previous efforts lacked (*20*).

It’s vital to acknowledge the limitations of our comparison, conducted within a limited timeframe and with a single analyst. To thoroughly gauge the scalability and robustness of the automated method, expanding the evaluation is imperative. Involving a larger sample size and multiple analysts working across different shifts would better mirror real laboratory conditions. Expanding such studies would offer a more comprehensive assessment of the protocol’s performance, its capacity to handle increased workloads, and its ability to maintain consistency and reproducibility across varying conditions.

The developed automated sample preparation method for IgG glycan analysis reduced exposure to hazardous chemicals and offered a simplified workflow. Despite differences between the methods, the new automated method showed high precision and proved to be highly comparable to its manual counterpart.

## Data Availability

The data generated and analysed in the presented study are available from the corresponding author on request.
